# Platensimycin Activity against Mycobacterial β-Ketoacyl-ACP Synthases

**DOI:** 10.1371/journal.pone.0006306

**Published:** 2009-07-17

**Authors:** Alistair K. Brown, Rebecca C. Taylor, Apoorva Bhatt, Klaus Fütterer, Gurdyal S. Besra

**Affiliations:** 1 School of Biosciences, College of Life and Environmental Sciences, University of Birmingham, Edgbaston, Birmingham, United Kingdom; 2 School of Applied Sciences, Northumbria University, Newcastle upon Tyne, United Kingdom; University of Hyderabad, India

## Abstract

**Background:**

There is an urgent need for the discovery and development of new drugs against *Mycobacterium tuberculosis*, the causative agent of tuberculosis, especially due to the recent emergence of multi-drug and extensively-drug resistant strains. Herein, we have examined the susceptibility of mycobacteria to the natural product platensimycin.

**Methods and Findings:**

We have demonstrated that platensimycin has bacteriostatic activity against the fast growing *Mycobacterium smegmatis* (MIC = 14 µg/ml) and against *Mycobacterium tuberculosis* (MIC = 12 µg/ml). Growth in the presence of paltensimycin specifically inhibited the biosynthesis of mycolic acids suggesting that the antibiotic targeted the components of the mycolate biosynthesis complex. Given the inhibitory activity of platensimycin against β-ketoacyl-ACP synthases from *Staphylococcus aureus*, *M. tuberculosis* KasA, KasB or FabH were overexpressed in *M. smegmatis* to establish whether these mycobacterial KAS enzymes were targets of platensimycin. In *M. smegmatis* overexpression of *kasA* or *kasB* increased the MIC of the strains from 14 µg/ml, to 30 and 124 µg/ml respectively. However, overexpression of *fabH* on did not affect the MIC. Additionally, consistent with the overexpression data, *in vitro* assays using purified proteins demonstrated that platensimycin inhibited Mt-KasA and Mt-KasB, but not Mt-FabH.

**Significance:**

Our results have shown that platensimycin is active against mycobacterial KasA and KasB and is thus an exciting lead compound against *M. tuberculosis* and the development of new synthetic analogues.

## Introduction

**Figure 1:**
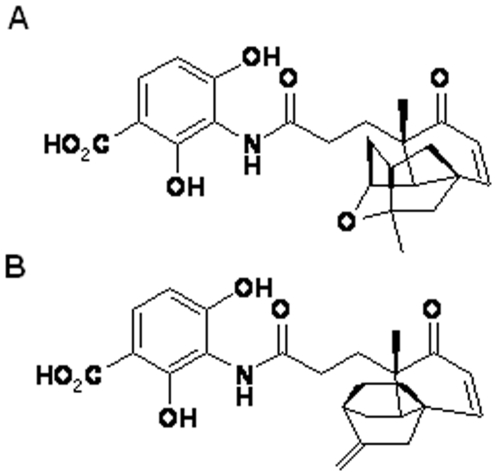
Structure of platensimycin (A) and platencin (B).

**Figure 2:**
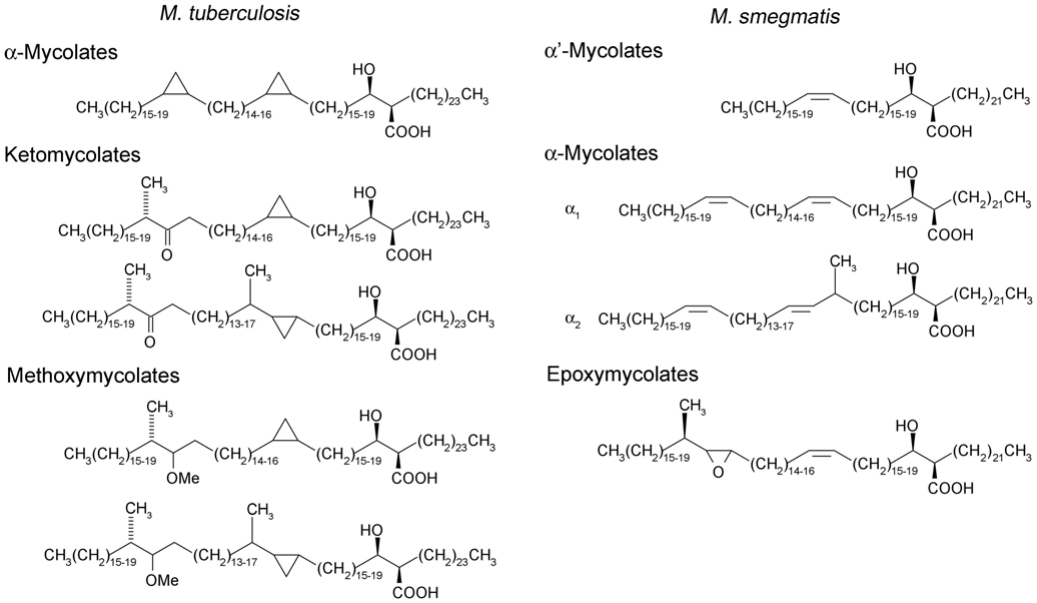
Structures of the major mycolic acids of *M. tuberculosis* and *M. smegmatis*.

Platensimycin (Figure 1A) is a secondary metabolite from *Streptomyces platensis* [1], [2], [3] which has been shown to possess potent anti-microbial activity against Gram-positive bacteria including methicillin-resistant *Staphylococcus aureus* (MRSA) and vancomycin-resistant *Enterococci* (VRE). The low mammalian cell toxicity and the lack of antifungal activity indicates that platensimycin acts selectively [3]. As a result platensimycin represents a promising new chemical class of antibiotics with *in vivo* activities of approximately 1 µg/ml towards *S. aureus*, *Enterococcus faecalis* and *Streptococcus pneumoniae* [3]. Platensimycin targets fatty acid biosynthesis in these species by inhibiting FabF and FabH, two β-ketoacyl-ACP synthases (KAS) of the bacterial multienzyme fatty acid synthase complex FAS-II [2], [3]. *Mycobacterium tuberculosis*, the causative agent of tuberculosis contains three distinct β-ketoacyl-ACP synthases, KasA, KasB and FabH [4], [5]. Of these, FabH acts as a pivotal link between a mammalian-like Fatty Acid Synthase I (FAS-I), a multifunctional enzyme that conducts *de novo* synthesis of C_16_ and C_26_ fatty acids, and Fatty Acid Synthase-II (FAS-II) a bacterial-type multi-enzyme complex that extends FAS-I products to long chain C_48–56_ fatty acids termed meromycolic acids. FAS-I derived C_26_ and meromycolic acids then undergo a Claisen-type condensation to form mycolic acids [6], [7], α-alkyl β-hydroxy fatty acids which are important and essential constituents of the mycobacterial cell wall (Figure 2). KasA and KasB are two distinct ketosynthases that are part of a core FAS-II complex which also includes a keto-reductase (FabG1, MabA), a multicomponent dehydratase (Rv0636+Rv0635 or Rv0637) and an enoyl reductase (InhA) [8], [9], [10], [11], [12], [13], [14], [15], [16]. This core complex is involved in a reductive cycle that elongates an acyl carrier protein (ACP)-bound acyl chain by iterative addition of two carbons using malonyl-ACP as a substrate, finally resulting in the formation of a meromycolate chain.

While *kasA* is an essential gene in mycobacteria [11], deletion of *Mycobacterium marinum kasB* [17] and *M. tuberculosis kasB* [10] resulted in viable strains that produced shorter meromycolate chains and were attenuated in macrophages and mice. In this study we have examined the whole cell susceptibility of *M. smegmatis* and *M. tuberculosis* to platensimycin. In addition, using discrete enzymes assays using purified Mt-KasA, Mt-KasB and Mt-FabH, we have established platensimycin as a promising lead compound for drug development.

## Results

### Whole cell activity of platensimycin against *Mycobacterium smegmatis*

**Figure 3:**
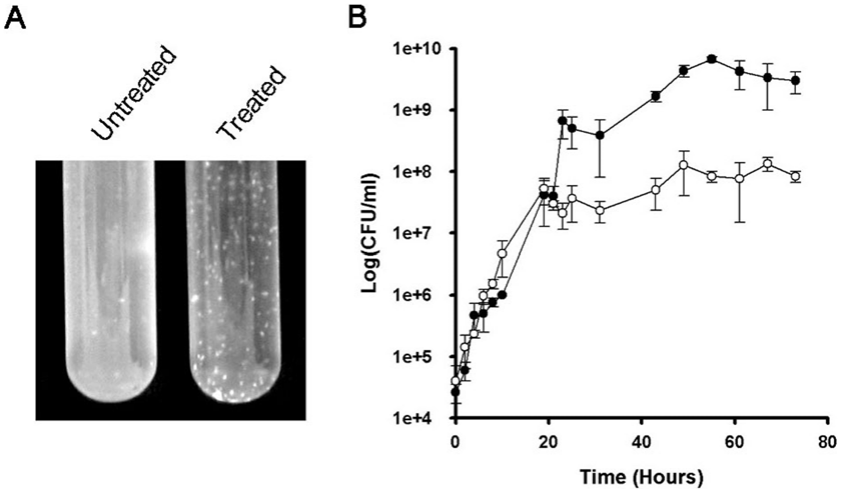
*In vivo* effect of platensimycin against *M. smegmatis*. *(A)* Clarification of cultures due to clumping and cellular lysis at time point 72 h. *(B)* Cultures were grown to an OD_600 nm_ of 0.4 upon which 14 µg/ml of platensimycin was added, samples were take over a 72 h period. Viable counts were calculated as *per* the methods where the mean CFU *per* millilitre from three independent experiments was calculated. •, *M. smegmatis*; ○, *M. smegmatis* + platensimycin.

Platensimycin has been previously shown to be an effective inhibitor of Gram-positive bacteria with MIC values as low as 1 µg/ml for *S. aureus*, *E. faecalis* and *S. pneumoniae* [3]. Platensimycin was initially tested for inhibitory properties against the non-pathogenic, fast growing *M. smegmatis* mc^2^155 which has been used in a number of studies as a surrogate for *M. tuberculosis*. The MIC_99_ of *M. smegmatis* in liquid medium was found to be 14 µg/ml (Table 1). We then monitored the growth of *M. smegmatis* in LB broth in the presence or absence of 14 µg/ml platensimycin for a period of 72 hours. While *M. smegmatis* grew normally in medium devoid of platensimycin, the culture in the medium containing platensimycin showed a decrease in OD_600_ values with time (data not shown) resulting in clumping after 24 hours of incubation (Figure 3A). Monitoring of viable colony forming units (CFU) demonstrated that the culture grown in the presence of platensimycin possessed a 2 log decrease in CFU (Figure 3B). The plateau shape observed with the treated cells, rather than a killing curve, would suggest that platensimycin is bacteristatic in nature. Further experimentation utilising cells exposed to platensimycin for 72 hours showed that after washing and re-inoculation into fresh media, treated cultures could be revived confirming that the antibiotic is bacteristatic against *M. smegmatis*.

**Table 1:** Influence of Mt-KasA, Mt-KasB and Mt-FabH overexpression on platensimycin in whole cell inhibition of *M. tuberculosis*, *M. smegmatis* and *M. bovis* BCG.

Strain	MIC_99_ (µg/ml)
***M. tuberculosis*** ** CDC1551**	12
***M. tuberculosis*** ** H37Rv**	12
***M. smegmatis***	14
***M. smegmatis*** ** pVV16**	14
***M. smegmatis*** ** pVV16-** ***KasA***	30
***M. smegmatis*** ** pVV16-** ***KasB***	124
***M. smegmatis*** ** pVV16-** ***KasAB***	126
***M. smegmatis*** ** pVV16-** ***FabH***	16
***M. bovis*** ** BCG**	>128
***M. bovis*** ** BCG pVV16**	>128
***M. bovis*** ** BCG pVV16-** ***KasA***	>128
***M. bovis*** ** BCG pVV16-** ***KasB***	>128
***M. bovis*** ** BCGΔ** ***KasB***	61

### Activity of platensimycin against slow growing mycobacteria

**Figure 4:**
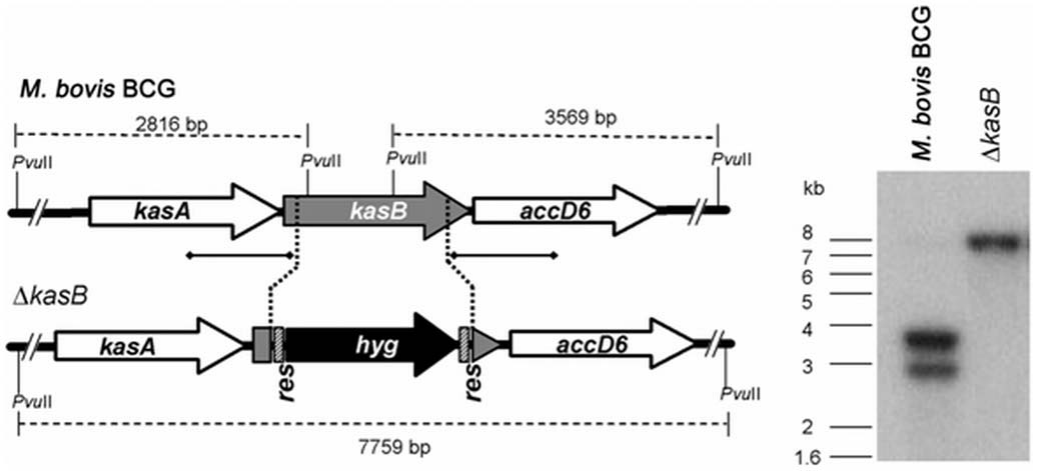
Generation of a *M. bovis* BCG *kasB* null mutant. Maps of the *kasB* region in the *M. bovis* BCG genome and its corresponding region in the conditional mutant Δ*kasB* are shown on the left, with the corresponding Southern blot on the right. The regions used as porbes are indicated by solid lines with square ends while the expected bands are indicated by broken lines (with sizes indicated). *hyg*, hygromycin resistance gene from *Streptomyces hygroscopicus*; *res*, γδ resolvase recognition sites.

To test the antimycobacterial potency of platensimycin against slow growing mycobacteria we first tested the activity of the antibiotic against *M. tuberculosis* CDC1551 and H37Rv. The MIC of platensimycin required to inhibit the growth of 99% of both *M. tuberculosis* strains on solid medium was 12 µg/ml (Table 1) indicating a comparable potency for this drug against this slow growing pathogen. Surprisingly, growth of the vaccine strain *M. bovis* BCG in the presence of platensimycin was different to that of *M. tuberculosis* and the strain grew normally in medium containing up to 128 µg/ml of platensimycin. In an effort to investigate the apparent resistance of BCG to platensimycin we sought to test the effects of increased membrane permeability by generating a *M. bovis* BCG Δ*kasB* mutant (Figure 4). It had been previously shown that a Δ*kasB* null mutant in *M. tuberculosis* synthesised shorter mycolic acids with almost a complete loss of *trans*-cyclopropanation of oxygenated mycolic acids that resulted in increased susceptibility to lipophilic antibiotics [10]. Interestingly, the *M. bovis* BCG Δ*kasB* (Table 1) mutant was sensitive to platensimycin (MIC 61 µg/ml) suggesting that the increased permeability in comparison to the parental *M. bovis* BCG strain has indeed increased the sensitivity of *M. bovis* BCG to platensimycin. However the high MIC of the mutant BCG strain in comparison to that in *M. tuberculosis* indicates that it is still unclear whether the resistance of BCG to platensimycin was solely due to decreased permeability to the drug.

### Platensimycin inhibits biosynthesis of fatty acids and mycolic acids

**Figure 5:**
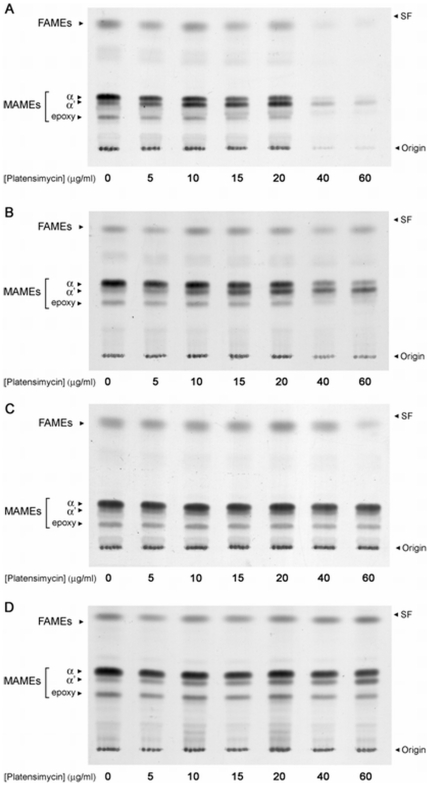
TLC-autoradiography of FAMEs and MAMEs from *M. smegmatis* strains overexpressing Mt-KasA, Mt-KasB and Mt-FabH following platensimycin treatment. Platensimycin (0–60 µg/ml) was titred into *M. smegmatis* cultures at an OD_600 nm_ of 0.4 prior to labelling with 1 µCi/ml [1,2-^14^C]acetate for 12 h. [^14^C]-FAMEs and MAMEs were extracted and resolved by TLC. An equivalent aliquot of the resulting solution of FAMEs and MAMEs was subjected to TLC using silica gel plates developed twice in petroleum ether-acetone (95∶5). Autoradiograms were produced by overnight exposure to Kodak X-Omat film to reveal [^14^C]labeled FAMEs and MAMEs. *(A) M. smegmatis* pVV16, *(B) M. smegmatis* pVV16-Mt-KasA, *(C) M. smegmatis* pVV16-Mt-KasB, and *(D) M. smegmatis* pVV16-Mt-KasAB.

**Figure 6:**
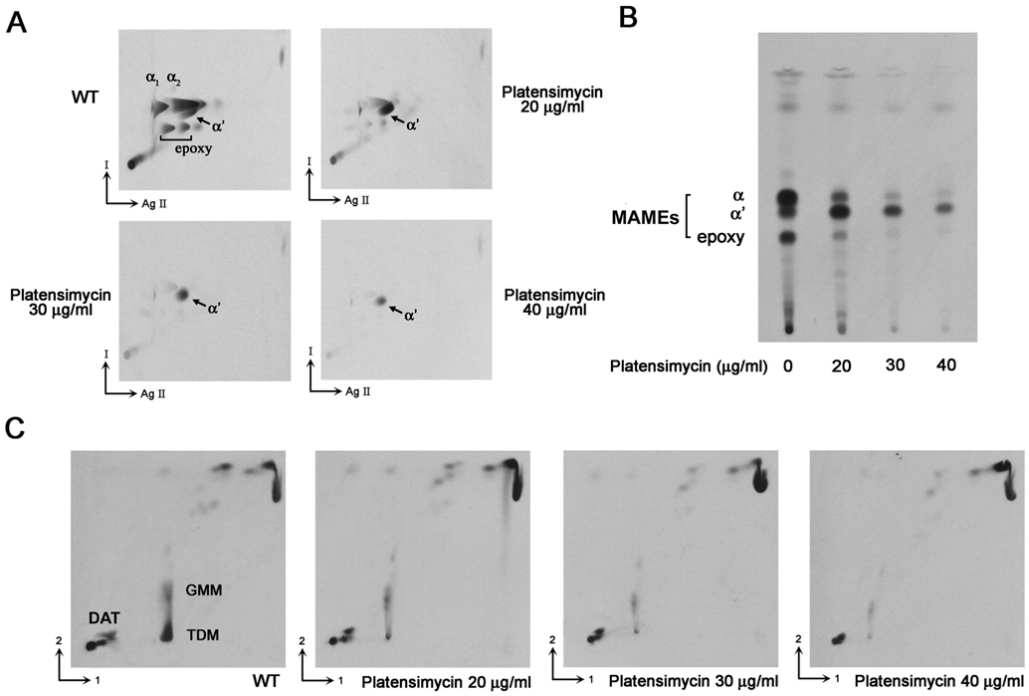
TLC-autoradiography of *M. smegmatis* lipid extracts and cell wall bound mycolates following platensimycin treatment. Platensimycin (0, 20, 30, 40 µg/ml) were added to *M. smegmatis* cultures at an OD_600 nm_ of 0.4 for 8 h prior to labelling with 1 µCi/ml [1,2-^14^C]acetate for 12 h. *(A)* 2D-Ag^2+^ TLC using silica gel plates developed twice in hexane-ethyl acetate (95∶5) (direction I) then thrice in petroleum ether-diethyl ether (85∶15) (direction Ag II). *(B)* Cell wall bound mycolate profiles were revealed following two developments in petroleum ether-acetone (95∶5). *(C)* [^14^C]-Apolar lipids were extracted and resolved by TLC; direction 1, chloroform-methanol-water (100∶14∶0.8); direction 2, chloroform-acetone-methanol-water (50∶60∶2.5∶3). DAT; diacyltrehalose, GMM; glucose monomycolate, TDM; trehalose dimycolate. Autoradiograms were produced by overnight exposure to film to reveal [^14^C]-labelled lipids.

To study the biochemical effects of platensimycin treatment, cultures of *M. smegmatis* mc^2^155 were metabolically labelled with [^14^C] acetate following exposure to platensimycin. Fatty acids and mycolic acids were extracted from [^14^C] labelled cells and methylated using phase-transfer catalysis and iodomethane. Extracts of total fatty acid methyl esters (FAMEs) and mycolic acid methyl esters (MAMEs) from untreated and platensimycin treated cultures (5–60 µg/ml) were analysed by TLC-autoradiography. Biosynthesis of fatty acids and α- and epoxy-mycolic acids (Figure 2) was significantly inhibited upon platensimycin treatment (20–40 µg/ml) (Figure 5A). Interestingly, an accumulation of α′-MAMEs was observed at lower concentrations (10–20 µg/ml) of platensimycin, similar to studies observed upon treatment of *M. smegmatis* with thiolactomycin (TLM), a known inhibitor of KasA and KasB (Figure 5A) [18]. The inhibition of fatty acids is in contrast to studies involving the FAS-II inhibitor isoniazid (INH) where inhibition of mycolic acid biosynthesis leads to an accumulation of fatty acids [19]. These results suggest that platensimycin also inhibits fatty acid biosynthesis *via* inhibition of mycobacterial FAS-I. Further analysis of the same samples by 2D-Ag^2+^ TLC reinforced these findings and revealed more clearly that synthesis of α (α_1_ and α_2_) and epoxy mycolic acids (Figure 6A) was abolished at lower concentrations, in comparison with the initial accumulation and then cessation α′-mycolic acid biosynthesis (Figure 6A). Futhermore, extracts of cell wall bound mycolic acids, afforded similar profiles upon platensimycin treatment (Figure 6B). In addition, analysis of [^14^C] labelled lipids extracted from platensimycin-treated cultures also revealed that the synthesis of mycolate containing lipids glucose monomycolate (GMM) and trehalose dimycolate (TDM) were reduced (Figure 6C; based on co-migration with authentic standards). The corresponding extracts did not show any platensimycin-derived effects on diacyltrehalose and glycopeptidolipid biosynthesis indicating that the inhibitory effect of platensimycin was specific to mycolate-containing glycolipids. These results demonstrated that platensimycin targeted fatty acid and mycolic acid biosynthesis in *M. smegmatis*.

### Platensimycin resistance of *M. smegmatis* strains overexpressing Mt-KasA, Mt-KasB or Mt-FabH

The use of gene overexpression to identify cellular targets of anti-mycobacterial drugs has been highly successful [20], [21], [22]. Given that the targets of platensimycin in other bacteria were β-ketoacyl synthases, we tested the effects of overexpression of Mt-KasA, Mt-KasB or Mt-FabH on platensimycin resistance in *M. smegmatis*. The three β-ketoacyl-ACP synthases, cloned into the *E. coli-Mycobacterium* shuttle vector pVV16 were introduced into *M. smegmatis* by eletroporation. Multiple copies and constitutive expression driven by the *hsp60* promoter ensures overexpression of the cloned genes in the host *Mycobacterium*. First, levels of each recombinant protein was assessed by western blot to confirm that any observed change in resistance could be attributed due to increased levels of the target protein (data not shown). Overexpression of Mt-KasA conferred a modest 2-fold increase in resistance to platensimycin, increasing the MIC from 14 to 30 µg/ml (Table 1). On the other hand, Mt-KasB overexpression resulted in a substantial 9-fold increase in the MIC to 124 µg/ml, respectively (Table 1). A combination of Mt-KasA/B overexpression failed to substantially enhance resistance to platensimycin further and possessed a MIC of 126 µg/ml. Interestingly, though platensimycin had a minimal effect on FabH of other bacteria [2], overexpression of Mt-FabH in theory should confer a small degree of resistance to platensimycin but no significant change was observed (Table 1). These observations were similar to resistance studies conducted with TLM and strains overexpressing Mt-FabH [23]. The nine-fold increase in resistance to platensimycin by *M. smegmatis* overexpressing Mt-*kasB* suggests that platensimycin preferentially targets KasB.

To further confirm the observed effects of overexpression on MICs, the ability of the recombinant *M. smegmatis* strains to incorporate [^14^C]-acetate into fatty acids and mycolic acids was examined. TLC analysis of FAMEs and MAMEs extracted from different strains treated with platensimycin revealed that whilst mycolic acid biosynthesis was only partially restored in the Mt-KasA overproducing strain, overexpression of either Mt-KasB or Mt-KasAB fully restored fatty acid and mycolic acid biosynthesis (Figure 5 B–D).

### Activity of platensimycin against *M. tuberculosis* β-ketoacyl-ACP synthases and FAS-I

To evaluate the effect of platensimycin on *in vitro* enzymatic activity, the impact upon [^14^C] malonate incorporation into fatty acids in cell free extracts of *M. smegmatis* enriched with FAS-I, and either purified Mt-KasA, Mt-KasB or Mt-FabH was assessed in discrete assays as described earlier [13], [21], [24]. In these assays [^14^C]malonyl-CoA is transacylated to AcpM via mtFabD prior to the addition of the relevant substrates (C_16_-AcpM and C_16_-CoA) and the enzyme of interest. Upon completion and termination of the experiment the radiolabelled acyl derivates are extracted using organic solvents. The assay was performed with a titre of platensimycin present in triplicate. The results were formulated into a graph where the 50% activity was calculated and noted as the IC_50_. Platensimycin was active against both Mt-KasA and Mt-KasB possessing IC_50_ values of 2 µg/ml (4.53 µM) and 4.2 µg/ml (9.51 µM), respectively (Table 2). These results are consistent with the *in vitro* inhibition of *S. aureus* FabF and *E. coli* FabF by platensimycin [2]. Interestingly, the IC_50_ values obtained with platensimycin are significantly lower than those obtained with another mycobacterial KAS inhibitor TLM (KasA = 20 µM, KasB 90 µM) [25]. While studies by Wang *et al.* [2] demonstrated that *S. aureus* FabH activity was inhibited by platensimycin (IC_50_ = 67 µM), consistent with our overexpression studies, Mt-FabH activity was insensitive to platensimycin (IC_50_>150 µg/ml, 340 µM) in comparison to Mt-KasA and Mt-KasB (Table 2).

**Table 2:** *In vitro* inhibition (IC_50_) of platensimycin against Mt-KasA, Mt-KasB, Mt-FabH and Ms-FAS-I and Cg-FAS-I.

	IC_50_ (µg/ml)
**Mt-KasA**	2 (0.1)
**Mt-KasB**	4.2 (0.1)
**Mt-FabH**	>150
**Ms-FAS-I**	12 (0.1)
**Cg-FAS-I**	6.5 (0.2)

Figures in brackets represent calculated standard error.

Interestingly, when crude cell free extracts of either *M. smegmatis* or the related *C. glutamicum* [26] were assayed for FAS-I activity, platensimycin inhibited FAS-I activity at an IC_50_ value of 12 µg/ml and 6.5 µg/ml, respectively (Table 2).

### Comparisons of *in silico* models of platensimycin-bound ketosynthases

Despite only a moderate level of sequence identity (∼36%) between Mt-KasB and *E. coli* FabF, the two enzymes display identical folds (Suppl. Figure 1A). To ascertain whether the active site of Mt-KasB would be compatible with the steric requirements of platensimycin, we generated a hypothetical structural model of platensimycin-bound Mt-KasB (Suppl. Figure 1B, C). Utilising the Mt-KasB (PDB code 2GP6) superposition with platensimycin-bound Ec-FabF (PDB code 2GFX). Subsequent conjugate-gradient energy minimization relieved mild steric clashes between protein and ligand and resulted, compared to ligand-free Mt-KasB, in minor to moderate shifts of side chains located within a 4 Å-radius of platensimycin (root mean square displacement 0.93 Å for 186 main and side chain atoms, maximum displacement 3.5 Å).

The model illustrates steric compatibility between platensimycin and the active site of Mt-KasB, but hints at subtle differences in protein-inhibitor interactions between Ec-FabF and Mt-KasB. The benzoic acid ring faces a structural environment that is virtually identical to that in Ec-FabF (Suppl. Figure 1B). However, the ketolide group would appear to be less exposed to solvent than it is in the Ec-FabF∶platensimycin complex (Figure S1 B,C). Indeed, the simulated complex structure suggests that the ketolide group makes close hydrophobic interactions with Met212 and Ile214 (Figure S1 B,C), contacts that are not seen in platensimycin-bound Ec-FabF, where these residues correspond to alanine side chain. Such differences provide scope for design efforts with the aim to enhance inhibitor affinity in a species-specific manner.

## Discussion

Mycolic acid biosynthesis is essential for mycobacterial survival and many antituberculosis drugs like isoniazid, ethionamide and thiolactomycin target enzymes of this exclusive pathway [8], [21]. The identification and functionality of novel lead antitubercular agents are essential in the present times due mainly to the emergence of MDR-TB [27] and more recently, XDR-TB [28]. In this worrying climate where untreatable strains of XDR-TB may become apparent the impetus for the discovery of new anti-tubercular agents becomes crucial. The discovery and characterization of new drugs targeting key enzymes involved in essential mycobacterial biosynthetic pathways is paramount but without the introduction of novel lead compounds, such as platensimycin antitubercular therapy will not proceed fast enough to cope with the increasing resistance observed today. Not only should drug development focus on the synthesis of active analogues of existing anti-mycobacterials, but also on identification of new classes of drugs, such as platensimycin.

Platensimycin, a natural product produced by *Streptomyces platensis* represents a new chemical class of antibiotics [3]. In this study we have shown that platensimycin is active against *M. tuberculosis* and through the use of the *M. smegmatis* surrogate system we were able to investigate and elucidate the mode of action of platensimycin against *Mycobacterium spp*. We observed inhibition of both mycobacterial FAS-I and FAS-II *in vitro* in cell free assays and resultant inhibition of both fatty acid and mycolic acid biosynthesis in whole cells treated with the antibiotic. Although, in *Mycobacterium* the fatty acyl-products of FAS-I provide primers for extension to meromycolate precursors of mycolic acids, the effects on FAS-II appear to be more complex than a simple deprivation of primer supply brought about *via* FAS-I inhibition. Therefore the combination of activity observed against both FAS-I and the FAS-II components KasA/KasB contributed to the inhibitory effect of platensimycin against *M. smegmatis*.

While from the point of view of drug development FAS-II inhibition, which causes cessation of the essential mycolic acids, is desirable, inhibition of FAS-I is not since mammalian fatty acid synthases are similar to FAS-I. Indeed, human and rat FAS-I were found to be inhibited *in vitro* by platensimycin [29]. However, platensimycin does not have the same effect when used on whole cells as it was previously demonstrated by the patent applicants Wang *et al.* [3] that platensimycin had low mammalian cell toxicity (IC_50_ of HeLa MTT >1000 µg/ml). Additionally, the authors also confirmed a lack of antifungal activity against *Candida albicans* which synthesises fatty acids *via* a type I fatty acid synthase (>64 µg/ml). Furthermore, our own studies with the related *C. glutamicum*, showed that while corynebacterial FAS-I activity was inhibited *in vitro* (Table 2), platensimycin failed to inhibit growth of cultures of *C. glutamicum* (data not shown). These findings suggested that, contrary to data obtained with *in vitro* inhibition assays with eukaryotic FAS-I, platensimycin selectively inhibits mycobacterial FAS-II *in vivo*.

Previous studies by Wang *et al.* [2] demonstrated that platensimycin poorly inhibited FabH in other bacteria. Interestingly, it was shown that platencin (Figure 1B) a related antibiotic was more active against *S. aureus* FabH (9.17 µM). Both platensimycin and platencin are structurally similar. Platensimycin contains a pentacyclic motif with a cyclic ether ring, whereas platencin contains a unique tetracyclic motif without the ether ring [2]. The chemical modification between platensimycin and platencin has been proposed to be responsible for the change in activity in *S. aureus* FabH. While platensimycin failed to inhibit mycobacterial Mt-FabH, it would be interesting to examine whether platencin is active against Mt-FabH. Altogether, the culture inhibition studies and *in vitro* assay data indicate that platensimycin targets Mt-KasA and Mt-KasB. Given that overexpression of Mt-KasA or Mt-KasB increased the MIC of the host *M. smegmatis* strain for platensimycin, it was surprising that platensimycin was relatively inactive against *M. bovis* BCG. Poor permeability may have been one of the factors responsible for the observed resistance. Indeed, deletion of *kasB* from *M. bovis* BCG resulted in a strain which was more permeable to lipophilic antibiotics [10] and thus sensitive to platensimycin (Table 1). However, it remains unclear whether poor permeability was the sole factor responsible for the resistance of BCG to the antibiotic. This data also suggests that in order to realise the full potential of this compound as an anti-tuberculosis agent, it is imperative that any future modifications to platensimycin include designs that render it more diffusible into the lipid-rich envelope of mycobacteria. Futhermore, *in silico* modelling of platensimycin-bound Mt-KasB suggested novel molecular interactions that were not seen in platensimycin-bound Ec-FabF. Such differences provide scope for design efforts with the aim to enhance inhibitor affinity in a species-specific manner. Our results highlight the potential of platensimycin as an inhibitor of the essential fatty acid and mycolic acid biosynthesis pathways in mycobacteria.

## Materials and Methods

### Plasmids, strains and DNA manipulation

The *Escherichia coli*-mycobacteria shuttle vector pVV16 (a gift from Varalakshmi Vissa, Colorado State University, CO, USA) containing the *hsp60* promoter and encoding a 6-histidine C-terminal tag was used for the over-expression of Mt-KasA and Mt-KasB. Mt-*kasA* PCR amplification was performed using the upstream primer 5′-gatcgatcaagcttgatgagtcagccttccaccg-3′ and the downstream primer 5′-gatcgatcaagcttgtaacgcccgaaggcaagc-3′, which contain HindIII and HindIII restriction sites, respectively (underlined). The 1251 bp PCR product was then digested with HindIII and ligated with similarly digested pVV16, giving rise to pVV16-Mt-KasA. Mt-*kasB* was cloned similarly using the upstream primer 5′-gatcgatccatatggatgggggtccccccgctt-3′ and the downstream primer 5′-gatcgatcaagcttgtaccgtccgaaggcgat-3′, which contain NdeI and HindIII restriction sites, respectively (underlined). The 1317 bp PCR product was then digested with NdeI and HindIII and ligated with similarly digested pVV16, giving rise to pVV16-Mt-KasB. Mt-FabH was cloned similarly using the upstream primer 5′-gatcgatccatatggtaccgtccgaaggcgat-3′ and the downstream primer 5′-gatcgatcaagcttacccttcggcattcgca-3′, which contain NdeI and HindIII restriction sites, respectively (underlined). The 1033 bp PCR product was then digested with NdeI and HindIII and ligated with similarly digested pVV16, giving rise to pVV16-Mt-FabH. The pVV16-KasAB was constructed using the Mt-*kasA* upstream primer and the Mt-*kasB* downstream primer, the 2623 bp PCR product was then digested with NdeI and HindIII and ligated with similarly digested pVV16, giving rise to pVV16-Mt-KasAB. The *kasB*-knockout phage phAE404 [10] was utilized to construct a *kasB* deletion in *M. bovis* BCG. Specialized transduction was performed as described in Bardarov *et al.* [30]. The validity of the *M. bovis* BCGΔ*kasB* was confirmed by Southern blot analysis (Figure 4). The coding sequences of all the recombinant genes were verified by DNA sequencing.

### Whole cell effects of platensimycin on *Mycobacterium spp*

*M. tuberculosis* CDC1551 was grown in 7H9 broth supplemented with 10% OADC enrichment and 0.05% Tween-80 to OD_600_ of 0.4. Following serial 10 fold dilutions, 20 ml of each dilution was spotted on 7H10 agar plates containing 0–128 µg/ml platensimycin. The minimum concentration of platensimycin required to inhibit growth of single colonies was noted as the minimum inhibitory concentration (MIC).

*M. smegmatis*-pVV16 and overexpression strains were grown in Luria-Bertani Broth (LB) (Difco) with 25 µg/ml kanamycin and 0.05% Tween 80 at 37°C to an optical density of 600 nm (OD_600_) of 0.25. A 10 ml culture was aliquoted and platensimycin added at the MIC of 15 µg/ml. The OD_600_ was recorded over 72 h and 100 µl samples were taken periodically and stored at 4°C for viable count analysis. After 72 h the cells were pelleted by centrifugation and washed with 8 ml of PBS buffer to remove platensimycin and the pellet resuspended in fresh LB media. The OD was recorded over 55 h and 100 µl samples taken and viable counts determined at each time point [31]. Briefly, the 100 µl samples were serially diluted to 10^−7^ and 10 µl samples, in triplicate, were spotted on to LB selective agar thrice. Following incubation at 37°C, the colonies were counted and converted into colony forming units (CFU) (CFU/ml). The MIC_99_ of platensimycin against *M. smegmatis* and *M. bovis* BCG were calculated by Alamar Blue testing as previously described [32]. Briefly, 200 µl of sterile deionized water was added to all outer-perimeter wells of a sterile 96-well plate (Corning Incorporated, Corning, NY, USA) to minimize evaporation of the medium in the test wells during incubation. The wells in rows B to G in columns 3 to 11 received 100 µl of 7H9 medium containing 25 µg/ml kanamycin, 50 µg/ml hygromycin and ADC (Beckton Dickinson, Sparks, MD). Platensimycin was added to rows B–G followed by 1∶2 serial dilutions across the plate to column 10, and 100 µl of excess medium was discarded from the wells in column 10. A bacterial culture (100 µl) was added to the wells in rows B to G in columns 2 to 11, where the wells in column 11 served as drug-free controls. The plates were sealed with parafilm and were incubated at 37°C for 24 h for *M. smegmatis* strains or 5 days for *M. bovis* BCG strains. A freshly prepared 1∶1 mixture of Alamar Blue (Celltiter-Blue™, Promega Corp, Madison, WI, USA) reagent and 10% Tween® 80 (50 µl) were added to well B11. The plates were reincubated at 37°C for 24 h. The cell viability assay was carried out as per the manufacturer's protocol followed by MIC_99_ calculations.

### Determination of the *in vivo* effects of platensimycin on cell envelope lipid synthesis

*M. smegmatis* cultures were grown to an OD_600 nm_ of 0.4 in the presence of 0.25% Tween 80 in Sautons medium at 37°C. Platensimycin was added at various concentrations followed by incubation at 37°C for 16 h for *M. bovis* BCG and 8 h for *M. smegmatis* at which point 1 µCi/ml [1,2-^14^C]acetate (57 mCi/mmol, GE Healthcare, Amersham Bioscience) was added to the cultures. The *M. bovis* BCG and *M. smegmatis* cultures were further incubated at 37°C for 24 h and 12 h, respectively. The [^14^C] labelled cells were harvested by centrifugation at 2000×*g*, washed with PBS and processed as described below.

The [^14^C] labelled cells were initially resuspended in CH_3_OH/0.3% NaCl (2 ml, 100∶10, v/v) and mixed with 1 ml of petroleum ether (60–80°C) for 15 min. The upper petroleum ether layer was removed and a further 1 ml of petroleum ether added, followed by further mixing for 15 min. The petroleum ether extracts were combined and evaporated under nitrogen using a heating block. The dried apolar lipid extract was resuspended in 200 µl of CH_2_Cl_2_ prior to thin-layer chromatography (TLC) and autoradiography [33]. Polar lipids were extracted by the addition of CHCl_3_/CH_3_OH/0.3% NaCl (2.3 ml, 9∶10∶3, v/v/v) to the lower methanolic saline phase and mixed for 1 h. The mixture was centrifuged and the pellet re-extracted twice with CHCl_3_/CH_3_OH/0.3% NaCl (750 µl, 5∶10∶4, v/v/v). CHCl_3_ (1.3 ml) and 0.3% NaCl (1.3 ml) were added to the combined extracts and the mixture centrifuged. The lower layer containing the polar lipids recovered and dried. The polar lipid extract was resuspended in CHCl_3_/CH_3_OH (2∶1, v/v). The apolar lipid extract (50,000 cpm) was applied to the corners of 6.6×6.6 cm plates of silica gel 60 F_254_ (Merck 5554) TLC plates. The plates were then developed using direction 1, chloroform-methanol-water (100∶14∶0.8, v/v/v) and direction 2, chloroform-acetone-methanol-water (50∶60∶2.5∶3, v/v/v/v) to separate [^14^C]-labelled lipids (TDM and glucose monomycolate [GMM]). Lipids were visualized by autoradiography by overnight exposure of Kodak X-Omat AR film to the TLC plates to reveal [^14^C]labelled lipids and compared to know standards [33].

### Determination of the *in vivo* effects of platensimycin on mycolic acid synthesis

The delipidated cells and whole cell pellets were similarly subjected to alkaline hydrolysis using 5% aqueous tetrabutylammonium hydroxide (TBAH) at 100°C overnight, followed by the addition of 4 ml of CH_2_Cl_2_, 500 µl of CH_3_I, 2 ml of water, followed by mixing for 30 min. The upper aqueous phase was discarded following centrifugation and the lower organic phase washed thrice with water and evaporated to dryness. The resulting FAMEs and MAMEs were dissolved in diethyl ether and insoluble residues removed by centrifugation. The etheral solution was evaporated to dryness and re-dissolved in 200 µl of CH_2_Cl_2_. Equivalent volumes of the resulting solution of FAMEs and MAMEs were subjected to TLC using silica gel plates (5735 silica gel 60F_254_; Merck, Darmstadt, Germany), developed in petroleum ether-acetone (95∶5). Autoradiograms were produced by overnight exposure of Kodak X-Omat AR film to the plates to reveal [^14^C]-labelled FAMEs and MAMEs. Ag^2+^-TLC was performed as described previously using Ag^2+^-impregnated TLC plates developed twice in direction I, hexane-ethyl acetate (95∶5, v/v), and then thrice in direction II, petroleum ether-acetone (85∶15, v/v) [34].

### Determination of the *in vitro* effects of platensimycin using crude cell-free extracts and purified proteins Mt-KasA, Mt-KasB and Mt-FabH

FAS-I extracts from *M. smegmatis* and *C. glutamicum* were prepared as described previously [35]. FAS-I experiments were conducted as described [18] using the 40–80% ammonium sulfate fraction [34]. Briefly, platensimycin was titred (0.1–150 µg/ml) into the standard reaction as follows: 100 mM potassium phosphate pH 7.0, 5 mM EDTA, 5 mM dithiothreitol, 300 µM acetyl-CoA, 100 µM NADPH, 100 µM NADH, 1 µM flavin mononucleotide, 500 µM α-cyclodextrin, 20 µM malonyl-CoA, 100,000 cpm of [2-^14^C] malonyl-CoA, and 100 µl of the cytosolic enzyme preparation (1 mg of protein) in a total volume of 500 µl. Reactions were performed in triplicate at 37°C for 1 h and terminated by the addition of 500 µl of 20% potassium hydroxide in 50% methanol at 100°C for 30 min. Following acidification with 300 µl of 6 M HCl, the resultant [^14^C]-labelled fatty acids were extracted three times with petroleum ether. The organic extracts were pooled, washed once with an equal volume of water, and dried in a scintillation vial prior to scintillation counting using 5 ml of EcoScintA (National Diagnostics, Hull, U.K.).

Mt-KasA, Mt-KasB and Mt-FabH proteins were purified and assayed as described previously [21], [24]. Briefly, platensimycin was titred (0.1–150 µg/ml) into the standard reaction as follows; Holo-AcpM (40 µg) was incubated on ice for 30 min with β-mercaptoethanol (0.5 µmol) in a total volume of 40 µl. [2-^14^C]malonyl-CoA (100, 000 cpm, 6.78 nmol, 1.66 kBq; Amersham), Mt-FabD (40 ng) and 25 µl of 1 M potassium phosphate buffer, pH 7.0, were added followed by incubation at 37°C for 30 min. C_16_-AcpM/holo-AcpM heterogeneous mix (22.5 µg) was added to obtain a final volume of 89 µl. Mt-KasA or Mt-KasB (0.25 µg) was added to initiate the reaction and was held at 37°C for 1 h. The reaction was quenched by the addition of 2 ml of a NaBH_4_ reducing solution (5 mg/ml NaBH_4_ in 0.1 M K_2_HPO_4_, 0.4 M KCl and 30% (v/v) THF). The reaction was held at 37°C for a further 1 h followed by two extractions with 2 ml of water-saturated toluene. The combined organic phases were pooled and washed using 2 ml of toluene-saturated water. The organic layer was removed and dried under a stream of nitrogen in a scintillation vial. The [^14^C_18_]-1,3-diol product was then quantified by liquid scintillation counting using 5 ml of EcoScintA (National Diagnostics, Hull, UK).

The activity of Mt-FabH was determined as previously described [24]. Platensimycin was titred (0.1–150 µg/ml) into the standard reaction as follows. The assays contained 50 µM holo-ACP/AcpM, 1 mM β-mercaptoethanol, 0.1 M sodium phosphate buffer, pH 7.0, 50 µM malonyl-CoA, 45 nCi of [2-^14^C]malonyl-CoA (100, 000 cpm, 6.78 nmol, 1.66 kBq; Amersham), 12.5 µM acyl-CoA primer, and Mt-FabD (0.3 µg of protein) in a volume of 50 µl and incubated at 37°C for 30 min. The reaction was initiated by the addition of 0.5 µg of Mt-FabH followed by incubation at 37°C for 40 min. The Mt-FabH assays were quenched and processed as described earlier for the Mt-KasA/B assays.

## Supporting Information

Figure S1 (1.05 MB DOC)
