# Supplementary material for: Platensimycin Activity against Mycobacterial β-Ketoacyl-ACP Synthases
Source: PLoS One. 2009 Jul 17;4(7):e6306. doi: 10.1371/journal.pone.0006306 (PMC2707616; doi:10.1371/journal.pone.0006306)

**Figure S1** **Comparison of KasB with *E. coli* FabF and simulated KasB:platensimycin complex.**

The structures of *E. coli* FabF_C163Q_ bound to platensimycin (PDB code 2GFX) and Mt-KasB (PDB code 2GP6) were superimposed using secondary structure matching, followed by 200 cycles of conjugate-gradient energy minimization of the (simulated) Mt-KasB:platensimycin complex using CNS 1.2 (Brunger AT, *Nat Protoc* **2**:2728-2733). *(A)* Ribbon diagram displaying Mt-KasB (blue) and *E. coli* FabF (grey) with platensimycin shown as sticks (magenta). *(B, C)* Two orthogonal views of the simulated Mt-KasB:platensimycin complex of Mt-KasB with carbon atoms of the experimental FabF and simulated Mt-KasB complex structures in grey and blue, respectively. Residue identifiers refer to Mt-KasB.

**Figure S1**


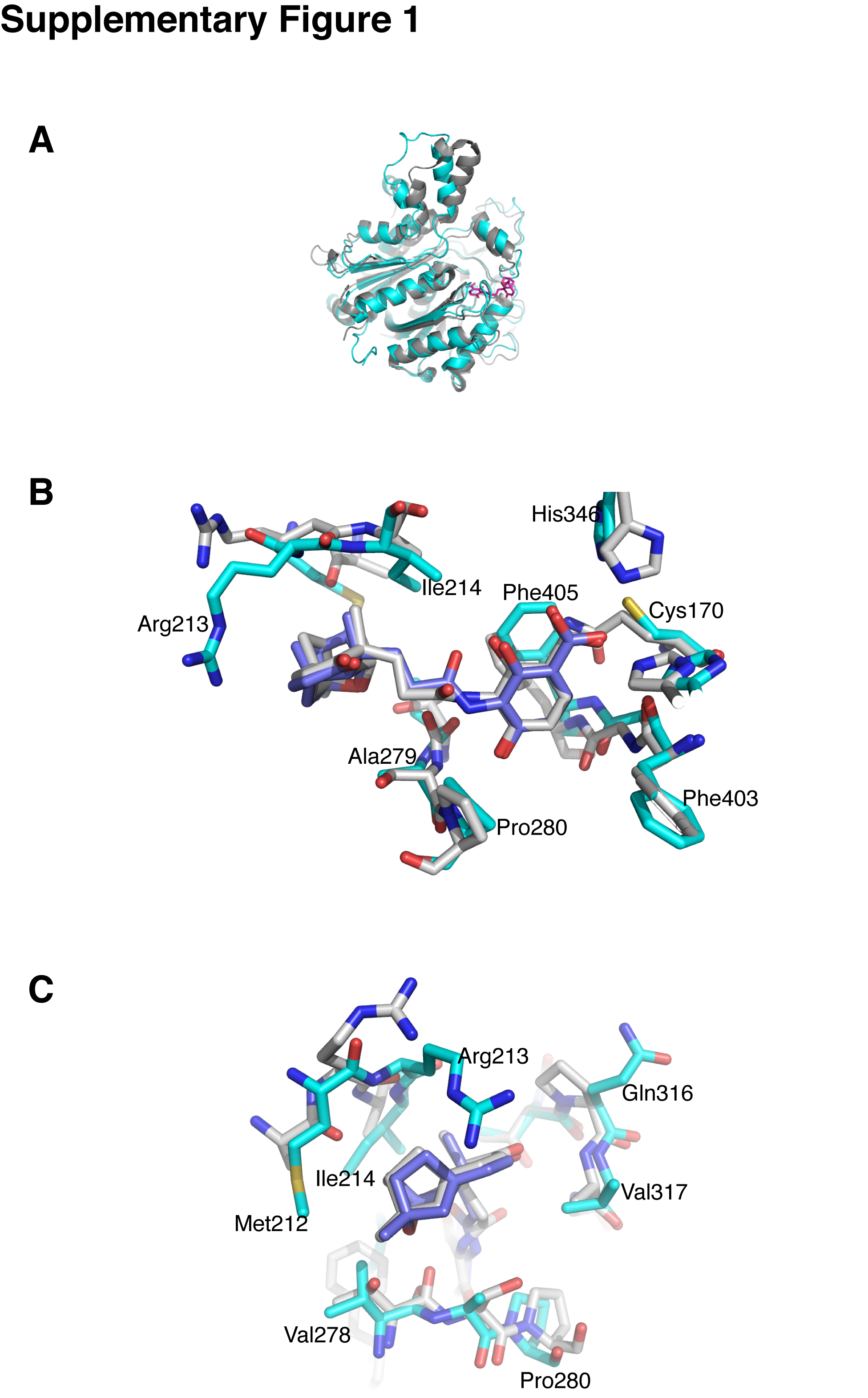

Supplement: Figure S1 — (1.05 MB DOC) [file pone.0006306.s001.doc]
